# Sleepiness in pregnancy and adverse perinatal outcomes

**DOI:** 10.1002/pmf2.70234

**Published:** 2026-01-21

**Authors:** Rachel Friedlander, Xiaoning Huang, Phyllis Zee, Sadiya S. Khan, Philip Greenland, Francesca L Facco, Judith Chung, William A. Grobman, Shalini Manchanda, Uma M. Reddy, George R. Saade, Robert M. Silver, Lynn M. Yee

**Affiliations:** ^1^ Department of Obstetrics and Gynecology Northwestern University Feinberg School of Medicine Chicago Illinois USA; ^2^ Department of Medicine Northwestern University Feinberg School of Medicine Chicago Illinois USA; ^3^ Department of Neurology Northwestern University Feinberg School of Medicine Chicago Illinois USA; ^4^ Department of Preventive Medicine Northwestern University Feinberg School of Medicine Chicago Illinois USA; ^5^ Department of Obstetrics and Gynecology University of Pittsburgh Pittsburgh Pennsylvania USA; ^6^ Department of Obstetrics and Gynecology UC Irvine Health Orange California USA; ^7^ Department of Obstetrics and Gynecology Brown University Providence Rhode Island USA; ^8^ Department of Medicine Indiana University School of Medicine Indianapolis Indiana USA; ^9^ Department of Obstetrics and Gynecology Columbia University New York New York USA; ^10^ Department of Obstetrics and Gynecology Eastern Virginia Medical School Norfolk Virginia USA; ^11^ Department of Obstetrics and Gynecology University of Utah Health Salt Lake City Utah USA

**Keywords:** gestational diabetes, hypertensive disorders of pregnancy, preterm birth, sleep disorders, sleepiness, small for gestational age birth

## Abstract

**Background:**

Sleep disorders in pregnancy have been associated with adverse pregnancy outcomes including hypertensive disorders of pregnancy, preterm birth, and gestational diabetes mellitus, yet these findings are largely from retrospective studies. Using data from a prospective study that used validated tools to assess sleepiness in pregnancy, we aimed to assess the association of sleepiness in pregnancy with adverse pregnancy outcomes.

**Methods:**

In this secondary analysis of data from a large, diverse prospective cohort of nulliparous individuals conducted at eight United States medical centers, those with self‐reported sleep data were included. Sleepiness was evaluated in early (6–13 weeks) and mid‐pregnancy (22–29 weeks) via the Epworth Sleepiness Scale, a validated tool to assess daytime sleep propensity (i.e., the ability to fall and stay asleep). Those with scores ≤10 were categorized as having no sleepiness (referent), 11–15 moderate sleepiness, and 16–24 severe sleepiness. The outcomes were hypertensive disorders of pregnancy, gestational diabetes, preterm birth, and small‐for‐gestational‐age birth < 10 percentile. Multivariable logistic regression analysis adjusting for multiple potential confounders was performed to evaluate the associations of moderate and severe sleepiness with each outcome.

**Results:**

Of 6806 eligible participants who completed daytime sleepiness questionnaires at both time points, the mean score in early and mid‐pregnancy was 7.8 (standard deviation 4.1) and 6.5 (standard deviation 4.0), respectively. In early pregnancy, 20.0% and 4.3% of people reported moderate and severe daytime sleepiness, respectively; in mid‐pregnancy, 14.4% and 2.5% of people experienced moderate and severe sleepiness. Severe sleepiness in mid‐pregnancy was associated with gestational diabetes (7.1% severe sleepiness vs. 3.8% no sleepiness; adjusted odds ratio [aOR], 1.78; 95% confidence interval [CI], 1.01–3.12) and small‐for‐gestational‐age birth (14.8% severe sleepiness vs. 10.3% no sleepiness; aOR, 1.35; 95% CI, 1.06–1.71).

**Conclusions:**

Severe daytime sleepiness during mid‐pregnancy, a symptom reflective of sleep quality, length, and often sleep disordered breathing, is associated with gestational diabetes and small‐for‐gestational‐age birth, suggesting a potential relationship with maternal glucose metabolism and placental function.

## 1 INTRODUCTION

Sleep disorders in pregnancy, such as insomnia and restless legs syndrome, are associated with adverse pregnancy outcomes, including hypertensive disorders of pregnancy (HDP), preterm birth (PTB), and gestational diabetes mellitus (GDM) [1, 2]. It has also been established that sleep disordered breathing, including obstructive sleep apnea, is associated with HDP and GDM [3].

Studies evaluating the association of daytime sleepiness, or the daytime propensity to fall asleep, with adverse pregnancy outcomes are limited to retrospective studies that lack the use of validated tools. The Epworth Sleepiness Scale is validated as a measurement of excessive daytime sleepiness in non‐pregnant individuals. One study using the Epworth Sleepiness Scale found a nearly sevenfold increased odds of GDM for people with excessive daytime sleepiness. However, these results were from a single study with a small sample size and at a single site [4].

Thus, we aimed to assess the association of sleepiness in pregnancy with adverse pregnancy outcomes in a large, multicenter, diverse prospective cohort of nulliparas.

## 2 METHODS

This is a secondary analysis of data from the Nulliparous Pregnancy Outcomes Study: Monitoring New Mothers‐to‐be (nuMoM2b), a prospective observational study conducted at eight United States medical centers from 2010 to 2013 that aimed to evaluate adverse pregnancy outcomes in a large, diverse cohort of nulliparous individuals [5]. Participants with self‐reported sleep data, but who did not have chronic hypertension or pregestational diabetes, were included in this analysis.

Sleepiness was evaluated via the Epworth Sleepiness Scale, a validated tool to assess excessive daytime sleepiness or sleep propensity (i.e., the ability to fall asleep). Those with scores ≤10 were categorized as having no sleepiness (referent), 11–15 as having moderate sleepiness, and 16–24 as having severe sleepiness. The Epworth Sleepiness Scale was conducted at two time points: 6–13 weeks (referred to as “early pregnancy”) and 22–29 weeks (referred to as “mid‐pregnancy”).

The pregnancy outcomes assessed were HDP, GDM, PTB, and small‐for‐gestational‐age (SGA) birth < 10th percentile. HDP was classified using the extant American College of Obstetrician and Gynecologists criteria for the spectrum of high blood pressure due to pregnancy, including preeclampsia, gestational hypertension, or eclampsia. GDM was defined based on clinical diagnosis using glucose tolerance testing results. PTB was defined as birth prior to 37 weeks and 0 days. Finally, SGA was defined as neonatal weight <10 percentile using the Alexander birthweight norms curve. Outcomes were abstracted by trained personnel from medical records according to standard obstetric definitions [5]. Multivariable logistic regression analysis was performed to evaluate the associations of moderate and severe sleepiness with each outcome. Multivariable models were adjusted for early pregnancy maternal age, self‐identified racial and ethnic identity as a social construct, insurance, early pregnancy body mass index, and current smoking within the prior three months.

Two sensitivity analyses were conducted. First, we performed a sensitivity analysis that included “high risk for sleep disordered breathing” as a covariate in the multivariable model as excessive daytime sleepiness is a common symptom in the setting of obstructive sleep apnea. High risk for sleep disordered breathing was measured by the Berlin Questionnaire for Sleep Apnea, a 10‐question self‐administered questionnaire screening for obstructive sleep apnea in which people are classified as high risk based on responses to questions about snoring, sleepiness, hypertension, and body mass index >30. Participants were classified as high risk based on scoring positively in two out of the three standardized symptom categories. Second, given the potential confounding nature of maternal anemia, a diagnosis that can be associated with sleepiness and SGA, we additionally performed a sensitivity analysis in which anemia at each time point, defined from chart review and as a self‐reported medical condition, was included in the adjusted model. Both obstructive sleep apnea and anemia were deemed appropriate as covariates as they are both associated with sleepiness and adverse pregnancy outcomes [6, 7].

## 3 RESULTS

In this cohort, there were 6806 eligible participants who completed sleep questionnaires at both time points. The average sleepiness score in early and mid‐pregnancy was 7.8 (standard deviation 4.1) and 6.5 (standard deviation 4.0), respectively. In early pregnancy, 20.0% and 4.3% of people experienced moderate and severe sleepiness, respectively. In mid‐pregnancy, 14.4% and 2.5% of people experienced moderate and severe sleepiness, respectively. Of those with moderate sleepiness in early pregnancy, 63.4% had no sleepiness in mid‐pregnancy and 31.6% had moderate sleepiness in mid‐pregnancy. Of those with severe sleepiness in early pregnancy, 41.2% had no sleepiness in mid‐pregnancy and 39.5% had moderate sleepiness in mid‐pregnancy. Those with severe sleepiness were more likely to be younger, non‐Hispanic White, privately insured, and a never smoker (Table 1).

**TABLE 1:** Baseline characteristics at first study visit and level of sleepiness.

	Total	No sleepiness	Moderate sleepiness	Severe sleepiness	
	*N* = 6806 100%	*N* = 5151 75.7%	*N* = 1359 20%	*N* = 296 4.3%	*p* value
Maternal age	27.3 (5.5)	27.5 (5.4)	26.9 (5.6)	25.5 (5.8)	<0.001
Self‐reported maternal race and ethnicity					<0.001
Non‐Hispanic White	64.8%	66.3%	62.2%	50.7%	
Non‐Hispanic Black	11.0%	9.6%	13.6%	23.0%	
Hispanic	15.1%	14.9%	15.0%	17.6%	
Asian	4.2%	4.3%	4.0%	3.0%	
Other	4.9%	4.8%	5.2%	5.7%	
Sources of payment					<0.001
Public insurance	22.6%	21.9%	23.3%	32.7%	
Private insurance	56.5%	57.1%	56.0%	49.0%	
Self‐pay or other sources	20.8%	21.0%	20.7%	18.4%	
Early pregnancy body mass index (kg/m^2^)	25.8 (5.9)	25.8 (5.8)	26.1 (6.1)	26.0 (6.3)	0.11
Underweight (<18.5)	2.3%	2.5%	1.7%	3.1%	0.28
Normal weight (18.5–24.9)	52.8%	53.1%	52.2%	49.5%	
Overweight (25–29.9)	25.1%	25.2%	24.4%	26.3%	
Obese (30–34.9)	11.4%	11.2%	12.4%	10.6%	
Morbidly obese (≥35)	8.4%	8.1%	9.3%	10.6%	
Nicotine exposure					<0.001
Never smoker	58.3%	57.2%	60.8%	67.2%	
Former smoker in the last 3 months	24.9%	26.1%	22.1%	17.2%	
Current smoker in the last 3 months	16.7%	16.7%	17.2%	15.5%	
Early pregnancy sleepiness score	7.8 (4.1)	6.0 (2.7)	12.5 (1.4)	17.5 (1.8)	<0.001
Mid‐pregnancy sleepiness score	6.5 (4.0)	5.6 (3.5)	9.0 (3.9)	11.2 (4.6)	<0.001
Mid‐pregnancy sleepiness level					<0.001
No sleepiness	83.1%	90.7%	63.4%	41.2%	
Moderate sleepiness	14.4%	8.4%	31.6%	39.5%	
Severe sleepiness	2.5%	0.9%	5.0%	19.3%	

*Note*: Table presents mean (standard deviation) for continuous variables and proportions for categorical variables. *p* values are based on *t*‐test for continuous variables and chi‐squared test for categorical variables.

In early pregnancy, moderate and severe sleepiness were not associated HDP, GDM, PTB, or SGA. Additionally, mid‐pregnancy moderate sleepiness was not associated with any of these adverse pregnancy outcomes. However, severe sleepiness in mid‐pregnancy was associated with GDM, with 7.1% of those with severe sleepiness having GDM versus 3.8% of those with no sleepiness. Findings persisted in adjusted models (adjusted odds ratio [aOR], 1.78; 95% confidence interval [CI], 1.01–3.12). Similarly, severe sleepiness in mid‐pregnancy was associated with SGA (14.8% severe sleepiness vs. 10.3% no sleepiness; aOR, 1.35; 95% CI, 1.06–1.71) (Table 2).

**TABLE 2:** Association of sleepiness and adverse pregnancy outcomes.

Total	No sleepiness	Moderate sleepiness	Severe sleepiness
**Early pregnancy (6–13 weeks)**			
*N* = 6806	*N* = 5151 (75.7%)	*N* = 1361 (20.0%)	*N* = 293 (4.3%)
HDP	13.8%	13.3% 0.92 (0.75, 1.13)	13.4% 0.94 (0.55, 1.61)
GDM	3.8%	4.2% 1.16 (0.74, 1.82)	4.1% 1.24 (0.70, 2.21)
PTB	6.8%	6.0% 0.80 (0.68, 0.94)	8.2% 1.17 (0.77, 1.78)
SGA	10.3%	10.8% 0.99 (0.84, 1.18)	9.8% 0.79 (0.57, 1.11)
**Mid‐pregnancy (22–29 weeks)**			
		*N* = 980 (14.4%)	*N* = 170 (2.5%)
HDP	14.0%	11.8% 0.80 (0.68, 0.95)	16.0% 0.95 (0.69, 1.33)
GDM	3.8%	4.0% 1.18 (0.80, 1.73)	7.1% **1.78 (1.01, 3.12)**
PTB	6.7%	6.3% 0.92 (0.73, 1.15)	8.9% 1.30 (0.76, 2.20)
SGA	10.3%	10.0% 0.92 (0.73, 1.16)	14.8% **1.35 (1.06, 1.71)**

*Note*: Data presented as % and adjusted odds ratio (95% confidence interval). Moderate sleepiness scores were defined as a score of 11–15; severe sleepiness scores were defined as 16–24. Multivariable models were adjusted for early pregnancy maternal age, race/ethnicity as a social construct, insurance, body mass index, and current smoking within the prior 3 months. The 95% confidence intervals are based on robust standard errors, which are clustered at the study sites. The sample excluded those with chronic hypertension and pregestational diabetes.The bolded values are the statistically significant adjusted odds ratios.

Abbreviations: HDP, hypertensive disorders of pregnancy; GDM, gestational diabetes; PTB, preterm birth (<37 weeks); SGA, small for gestational age status (<10 percentile).

Findings were similar when controlling for sleep disordered breathing in a sensitivity analysis (Table S4). Although the confidence interval crossed unity when controlling for obstructive sleep apnea, there was a <10% change in the estimated odds ratio of the association between mid‐pregnancy severe sleepiness and GDM (aOR, 1.71; 95% CI, 0.97–3.04). Similarly, mid‐pregnancy severe sleepiness remained significantly associated with SGA (aOR, 1.45; 95% CI, 1.16–1.81).

In the models in which maternal anemia was included as a potential confounder, findings were similar. Specifically, mid‐pregnancy severe sleepiness remained associated with SGA (aOR, 1.41; 95% CI, 1.07–1.85) and GDM (aOR, 1.93; 95% CI, 1.05–3.56) after accounting for anemia (Table S5).

## 4 DISCUSSION

In this study of nulliparous individuals undergoing prospective assessment of sleep characteristics and pregnancy outcomes, about a quarter of the population and a fifth of the population reported at least moderate sleepiness by early or mid‐pregnancy, respectively. The results demonstrate that severe sleepiness in mid‐pregnancy is significantly associated with both GDM and SGA, and results of sensitivity analyses indicate that these associations persist even when accounting for sleep‐disordered breathing and maternal anemia.

The relationship of sleep disorders to aberration of antenatal metabolism remains an active area of research. Previous studies of insomnia and restless legs syndrome during pregnancy suggested that these sleep conditions were not associated with GDM. In contrast, short sleep duration and later sleep midpoint have been shown to be risk factors for GDM in pregnant individuals [8]. Poor sleep quality and duration have been extensively studied in nonpregnant adults and has been linked to type 2 diabetes mellitus, likely due to neuroendocrine changes that decrease insulin sensitivity and increase appetite through decreased leptin and increased ghrelin levels [9]. Isolating the concept of sleepiness alone, which encompasses a diagnosis beyond sleep duration, as an independent risk factor for insulin resistance, is less well studied. A large, cross‐sectional study utilizing NHANES data found that diabetes was associated with excessive daytime sleepiness [10]. However, the directionality and the underlying mechanism of this association are areas for future work.

The association between severe sleepiness and SGA may be linked to uteroplacental insufficiency. Sleep disordered breathing and anemia have been studied to be associated with SGA [7, 11]. Notably, the relationship between sleepiness and SGA persisted even after controlling for these diagnoses in separate sensitivity analyses.

In summary, our findings highlight a notable prevalence of moderate to severe sleepiness in pregnancy and demonstrate its association with adverse outcomes, particularly GDM and SGA. The strengths of this study are that prospective data collection was used in a large, diverse cohort using validated sleep questionnaires, whereas previous studies had smaller sample sizes and were retrospective. One limitation of the current study is that the Epworth Sleepiness Scale has been validated in a nonpregnant population but is utilized in the current study for pregnant people. Additionally, given the exploratory nature of this analysis, the possibility of type 1 error exists.

Although the mechanisms linking sleepiness to these outcomes remain to be fully understood, our results suggest that sleepiness even after adjusting for potential confounders may serve as a distinct and underrecognized risk factor in pregnancy. Further research is needed to elucidate the biological pathways involved and to determine whether early identification and intervention could mitigate these risks.

## CONFLICT OF INTEREST STATEMENT

The authors declare no conflicts of interest.

## ETHICS STATEMENT

All participants provided written informed consent prior to participation. The study was approved by the institutional review boards at each site.

## Supporting information



Supporting Information

## Data Availability

The nuMoM2b Study (Nulliparous Pregnancy Outcomes Study: Monitoring Mothers‐to‐Be) dataset is available for download from the National Institutes of Health, *Eunice Kennedy Shriver* National Institute of Child Health and Human Development's Data and Specimen Hub (DASH) (https://dash.nichd.nih.gov/).
