# Supplementary material for: Sleepiness in pregnancy and adverse perinatal outcomes
Source: Pregnancy (Hoboken). 2026 Jan 21;2(1):e70234. doi: 10.1002/pmf2.70234 (PMC13344581; doi:10.1002/pmf2.70234)
Supplement: Supplementary file 1 [file PMF2-2-e70234-s001.docx]

| Table 3 Association of sleepiness and adverse pregnancy outcomes, unadjusted OR | | | | | | | |  |
| --- | --- | --- | --- | --- | --- | --- | --- | --- |
|  | | **Total** | | **Moderate Sleepiness** | | | **Severe Sleepiness** |  |
| Early pregnancy (6-13 weeks) | | | | | | | |  |
|  | N=6, 806 | | | | N= 1,361 (20.0%) | N= 293 (4.3%) | |  |
| HDP | | |  | 0.95 (0.76, 1.20) | | 0.97 (0.57, 1.64) | |  |
| GDM | | |  | 1.11 (0.70,1.77) | | 1.08 (0.60, 1.95) | |  |
| PTB | | |  | 0.87 (0.72, 1.04) | | 1.22 (0.78, 1.93) | |  |
| SGA | | |  | 1.04 (0.88, 1.24) | | 0.94 (0.64, 1.37) | |  |
| Mid-pregnancy (22-29 weeks) | | | | | | | |  |
|  | | |  | N= 980 (14.4%) | | N= 170 (2.5%) | |  |
| HDP | | |  | 0.82 (0.66, 1.03) | | 1.17 (0.89, 1.55) | |  |
| GDM | | |  | 1.06 (0.67, 1.69) | | 1.92 (1.04, 3.55) | |  |
| PTB | | |  | 0.94 (0.75, 1.17) | | 1.35 (0.82, 2.23) | |  |
| SGA | | |  | 0.97 (0.79, 1.19) | | 1.51 (1.11, 2.05) | |  |
| Data presented as % and unadjusted odds ratio (95% confidence interval)  HDP- Hypertensive disorders of pregnancy, GDM- Gestational diabetes, PTB-preterm birth (<37w), SGA- Small for gestational age status (<10%ile), aOR- adjusted odds ratio, CI- confidence interval   1. Moderate sleepiness scores were defined as a score of 11-15; Severe sleepiness score were defined as 16-24. 2. Multivariable models were adjusted for early pregnancy maternal age, race/ethnicity as a social construct, insurance, body mass index, and current smoking within the prior 3 months. 3. The 95% confidence intervals are based on robust standard errors, which are clustered at the study sites. 4. The sample excluded those with chronic hypertension and pregestational diabetes. | | | | | | | |  |

| Table 4 Association of sleepiness and adverse pregnancy outcomes, sensitivity analysis for sleep disordered breathing | | | | | | | |  |
| --- | --- | --- | --- | --- | --- | --- | --- | --- |
|  | | **Total** | | **Moderate Sleepiness** | | | **Severe Sleepiness** |  |
| Early pregnancy (6-13 weeks) | | | | | | | |  |
|  | N=6, 806 | | | | N= 1,361 (20.0%) | N= 293 (4.3%) | |  |
| HDP | | |  | 0.91 (0.74, 1.11) | | 0.93 (0.55, 1.58) | |  |
| GDM | | |  | 1.08 (0.69,1.70) | | 1.21 (0.69, 2.13) | |  |
| PTB | | |  | 0.80 (0.68, 0.94) | | 1.19 (0.78, 1.81) | |  |
| SGA | | |  | 0.99 (0.84, 1.17) | | 0.82 (0.58, 1.14) | |  |
| Mid-pregnancy (22-29 weeks) | | | | | | | |  |
|  | | |  | N= 980 (14.4%) | | N= 170 (2.5%) | |  |
| HDP | | |  | 0.77 (0.67, 0.90) | | 0.87 (0.63, 1.20) | |  |
| GDM | | |  | 1.15 (0.77, 1.72) | | 1.71 (0.97, 3.04) | |  |
| PTB | | |  | 0.90 (0.72, 1.13) | | 1.25 (0.73, 2.12) | |  |
| SGA | | |  | 0.93 (0.72, 1.20) | | 1.45 (1.16, 1.81) | |  |
| Data presented as % and adjusted odds ratio (95% confidence interval)  HDP- Hypertensive disorders of pregnancy, GDM- Gestational diabetes, PTB-preterm birth (<37w), SGA- Small for gestational age status (<10%ile), aOR- adjusted odds ratio, CI- confidence interval   5. Moderate sleepiness scores were defined as a score of 11-15; Severe sleepiness score were defined as 16-24. 6. Multivariable models were adjusted for early pregnancy maternal age, race/ethnicity as a social construct, insurance, body mass index, current smoking within the prior 3 months, and high risk for sleep disordered breathing. 7. The 95% confidence intervals are based on robust standard errors, which are clustered at the study sites. 8. The sample excluded those with chronic hypertension and pregestational diabetes. | | | | | | | |  |

| Table 5 Association of sleepiness and adverse pregnancy outcomes, sensitivity analysis for anemia | | | | | | | |  |
| --- | --- | --- | --- | --- | --- | --- | --- | --- |
|  | | **Total** | | **Moderate Sleepiness** | | | **Severe Sleepiness** |  |
| Early pregnancy (6-13 weeks) | | | | | | | |  |
|  | N=6, 806 | | | | N= 1,361 (20.0%) | N= 293 (4.3%) | |  |
| HDP | | |  | 0.92 (0.74, 1.13) | | 0.95 (0.55, 1.63) | |  |
| GDM | | |  | 1.16 (0.74,1.83) | | 1.26 (0.70, 2.27) | |  |
| PTB | | |  | 0.81 (0.68, 0.96) | | 1.19 (0.79, 1.81) | |  |
| SGA | | |  | 0.99 (0.84, 1.17) | | 0.81 (0.59, 1.11) | |  |
| Mid-pregnancy (22-29 weeks) | | | | | | | |  |
|  | | |  | N= 980 (14.4%) | | N= 170 (2.5%) | |  |
| HDP | | |  | 0.83 (0.68, 1.02) | | 1.02 (0.70, 1.48) | |  |
| GDM | | |  | 1.25 (0.82, 1.90) | | 1.93 (1.05, 3.56) | |  |
| PTB | | |  | 0.99 (0.76, 1.28) | | 1.45 (0.89, 2.37) | |  |
| SGA | | |  | 0.95 (0.75, 1.20) | | 1.41 (1.07, 1.85) | |  |
| Data presented as % and adjusted odds ratio (95% confidence interval)  HDP- Hypertensive disorders of pregnancy, GDM- Gestational diabetes, PTB-preterm birth (<37w), SGA- Small for gestational age status (<10%ile), aOR- adjusted odds ratio, CI- confidence interval   9. Moderate sleepiness scores were defined as a score of 11-15; Severe sleepiness score were defined as 16-24. 10. Multivariable models were adjusted for early pregnancy maternal age, race/ethnicity as a social construct, insurance, body mass index, current smoking within the prior 3 months, and anemia in early pregnancy or mid-pregnancy. 11. The 95% confidence intervals are based on robust standard errors, which are clustered at the study sites. 12. The sample excluded those with chronic hypertension and pregestational diabetes. | | | | | | | |  |
